# Gayet wernicke encephalopathy: Don’t miss this neuropsychiatric emergency!

**DOI:** 10.1192/j.eurpsy.2021.967

**Published:** 2021-08-13

**Authors:** S. Laroussi, K.S. Moalla, O. Hdiji, S. Sakka, S. Daoud, H. Hadjkacem, N. Farhat, C. Mhiri

**Affiliations:** Neurology, Habib Bourguiba Hospital, sfax, Tunisia

**Keywords:** Gayet Wernicke encephalopathy, thiamin, clinical symptoms, Radiologic features

## Abstract

**Introduction:**

Gayet Wernicke Encephalopathy (GWE) is a diagnostic and therapeutic neuropsychiatric emergency due to thiamin deficiency (vitamin B1).

**Objectives:**

The purpose of our work is to recall some clinical situations suspecting GWE, along with radiological and evolutionary profile.

**Methods:**

We conducted a retrospective study concerning patients who were hospitalized in the neurology department of Habib Bourguiba Hospital between 2013 and 2020 for management of GWE.

**Results:**

The median age of 7 patients was 39.57 years with sex ratio (H/F):1.33. The most common risk factor found is incoercible vomiting (5 patients), followed by chronic alcoholism (3 patients). Confusional state was the most frequent symptom found in 4 patients. The characteristic clinical triad of confusion, oculomotor disorders and ataxia was only found in 2 patients. Neuroimaging showed a typical aspect in 3 patients. The serum levels of thiamine were low in five patients and normal in two patients. After receiving parental than oral thiamin supplementation, three patients were independent after one month with a mRS score <3.

**Conclusions:**

GWE is an acute neuropsychiatric emergency. Chronic alcoholism is recognized as its most common cause. The clinical triad is not constantly present. MRI shows typically bilateral symmetrical hyperintensities in periaqueductal area, periventricular region, thalami and mammillary bodies. Thiamin level can be normal since it does not accurately represent body thiamine status or in case of mutations in a thiamine-transporter gene. Thiamine therapy is warranted if any component of the GWE triad is present in an appropriate clinical setting to prevent irreversible neurological sequelae.

